# The Effects of Preference for Information on Consumers’ Online Health Information Search Behavior

**DOI:** 10.2196/jmir.2783

**Published:** 2013-11-26

**Authors:** Yan Zhang

**Affiliations:** ^1^University of Texas at AustinAustin, TXUnited States

**Keywords:** preference for information, health information, consumer search behavior, search engines

## Abstract

**Background:**

Preference for information is a personality trait that affects people’s tendency to seek information in health-related situations. Prior studies have focused primarily on investigating its impact on patient-provider communication and on the implications for designing information interventions that prepare patients for medical procedures. Few studies have examined its impact on general consumers’ interactions with Web-based search engines for health information or the implications for designing more effective health information search systems.

**Objective:**

This study intends to fill this gap by investigating the impact of preference for information on the search behavior of general consumers seeking health information, their perceptions of search tasks (representing information needs), and user experience with search systems.

**Methods:**

Forty general consumers who had previously searched for health information online participated in the study in our usability lab. Preference for information was measured using Miller’s Monitor-Blunter Style Scale (MBSS) and the Krantz Health Opinion Survey-Information Scale (KHOS-I). Each participant completed four simulated health information search tasks: two look-up (fact-finding) and two exploratory. Their behaviors while interacting with the search systems were automatically logged and ratings of their perceptions of tasks and user experience with the systems were collected using Likert-scale questionnaires.

**Results:**

The MBSS showed low reliability with the participants (Monitoring subscale: Cronbach alpha=.53; Blunting subscale: Cronbach alpha=.35). Thus, no further analyses were performed based on the scale. KHOS-I had sufficient reliability (Cronbach alpha=.77). Participants were classified into low- and high-preference groups based on their KHOS-I scores. The high-preference group submitted significantly shorter queries when completing the look-up tasks (*P*=.02). The high-preference group made a significantly higher percentage of parallel movements in query reformulation than did the low-preference group (*P*=.04), whereas the low-preference group made a significantly higher percentage of new concept movements than the high-preference group when completing the exploratory tasks (*P*=.01). The high-preference group found the exploratory tasks to be significantly more difficult (*P*=.05) and the systems to be less useful (*P*=.04) than did the low-preference group.

**Conclusions:**

Preference for information has an impact on the search behavior of general consumers seeking health information. Those with a high preference were more likely to use more general queries when searching for specific factual information and to develop more complex mental representations of health concerns of an exploratory nature and try different combinations of concepts to explore these concerns. High-preference users were also more demanding on the system. Health information search systems should be tailored to fit individuals’ information preferences.

## Introduction

Searching for health information online is one of the most popular uses of the Web in the United States across all age groups [1]. When facing health threats, information seeking can enable patients to improve their ability to manage problems and make informed decisions [2] or to make psychosocial and emotional adjustments to illnesses [3]. In everyday life situations, information seeking is a means of gaining knowledge about health behaviors and disease prevention [1,4]. Whether and how users proceed with information seeking and their engagement in the activity, such as the selection of sources, the scope of sources investigated, the types and amount of information sought, and the depth of investigation are affected not only by their demographics (eg, age, gender, and socioeconomic status), knowledge levels (eg, computer and health literacy) and contextual factors (eg, the complexity of a health problem), but also by the individual’s personality characteristics, such as locus of control, self-efficacy, and preference for information [3,5-8].

As health care moves from a paternalistic model to a shared decision-making model [8], an unprecedented need is imposed on patients to acquire health-related information. As a result, preference for information, among various personality factors, has drawn much attention from researchers and medical practitioners alike [8,9]. Preference for information is an individual’s general tendency toward engaging in health information-seeking behavior. From a stress and coping perspective, it is an enduring personality trait that affects people’s use of information seeking as a means of coping with stressful health conditions [10]. Based on this trait, individuals can be classified into “monitors” and “blunters”. Monitors are people who are alert and sensitive to the environment and who actively scan it for information to help them cope with stress. Blunters are people who tend to avoid information or distract themselves from it. Miller’s Monitor-Blunter Style Scale (MBSS) helps identify monitors and blunters [11]. From the personal control perspective, preference for information (or informational involvement) and behavioral involvement are considered to be the two most common approaches for people to gain a sense of control and a belief that they can alter or affect outcomes. The Krantz Health Opinion Survey-Information Scale (KHOS-I) was developed to measure individuals’ informational involvement [12].

Empirical studies have provided evidence in support of the power of preference for information, measured by the MBSS, in predicting people’s information-seeking behavior in various contexts of patient-provider interactions, such as people undergoing cancer screening [13], women before gynecological surgery [14], patients in cancer treatments with a palliative intention [15], soldiers with combat-related post-traumatic stress disorder [16], and women with multiple sclerosis [17]. A common finding is that monitors, compared to blunters, have more doubts about medical procedures, desire more information, and ask more questions of providers. Consequently, monitors tend to have significantly more knowledge about medical procedures and their medical situation. The relationship between preference for information and information seeking seems to hold in stressful nonmedical situations as well. For example, Bar-Tal and Spitzer [18] found that, in stressful interpersonal conflict situations, undergraduate students with a monitoring style were more likely to seek information and support from others to solve problems.

Preference for information is also related to people’s interactions with written information. Examining the information needs of women with multiple sclerosis, Baker [17] found that more monitors than blunters rated as relevant a pamphlet providing disease-related information (fatigue or treatment for acute attacks), regardless of whether the information was general or specific. Koo et al [19] found that the monitoring versus the blunting style could predict patients’ interest in reading about and seeking written information concerning their prescription medicines, with monitors being more than twice as likely to be interested in reading such information.

Similarly, preference for information, measured by the KHOS-I, was found to predict people’s health-related information-seeking behavior. In an early study on college students who visited a college medical office with complaints such as headaches, colds, and flu, Krantz et al [12] found that participants who received higher KHOS-I scores asked more questions during the visit. Barsevick and Johnson [20] found that higher KHOS-I scores also predicted the number of questions that women undergoing a colposcopy asked their providers.

Preference for information is not only related to people’s information-seeking behavior, but also to their cognitive and emotional states. The monitoring style is often associated with higher concern and anxiety levels and higher demands for assurance. For example, Miller [13] found that, in cancer screening, patients with a monitoring coping style were more concerned and distressed about their cancer risk. Caldwell [21] found that patients who scored highly on the KHOS-I were more anxious in a preoperative setting than those who scored low, due to a tendency to focus on the negative aspects of the threatening situation. Mahler and Kulik [22] identified a similar correlation in male coronary-artery-bypass patients: those with higher KHOS-I scores experienced more social interaction and emotional problems and, as a result, desired more information to help reduce uncertainty and emotional arousal.

Not surprisingly, monitors and blunters are affected differently by information. Although the findings are not conclusive [23], it appears that individuals who prefer to have information and are given information tend to become less anxious, while those who have a low preference for information but are given information tend to become more anxious [13,24-29]. For example, Morgan et al [30] found that, before a colonoscopy, patients given information congruent with their coping style experienced significantly less self-reported anxiety after the information intervention and spent less time in recovery. In contrast, patients given information not congruent with their coping style maintained their pre-intervention anxiety level. Patients are also more likely to take action when information interventions are congruent with their dispositional preference for information. Williams-Piehota et al [31] found that when provided with detailed reassuring messages, monitors were more likely to obtain mammograms and, when provided with more concise and simple messages, they were less likely to obtain mammograms. In contrast, blunters were more motivated by the less detailed messages.

As reviewed, most studies on preference for information have focused on its relationship with information-seeking behavior in the context of patient-physician interaction or information interventions offered by providers (eg, number of questions asked and the amount of information sought), as well as its relationship with patients’ cognition (eg, uncertainty) and emotions (eg, anxiety, level of stress, and satisfaction with treatments). Few studies have examined whether it has an impact on general consumers’ health information search behavior—the behavior while interacting with search systems - for example, whether people with a higher preference for information explore more search results. There is also a lack of research on how this personality trait impacts the other two important aspects of an information search experience: people’s perceptions of search tasks (as representations of information needs) and their experience with search systems [32]. These are important research topics, as more than 80% of US adult Web users search online for health information and the need for a personalized health information search experience keeps increasing [33]. This study intends to fill these gaps by addressing three research questions:

1. Does the participants’ preference for information affect their behavior when they interact with search engines in completing look-up versus exploratory tasks?
2. Does preference for information affect participants’ perceptions of task difficulty, the mental effort required to complete the tasks, and satisfaction with their performance?
3. Does preference for information affect participants’ experience with search systems?

Knowledge gained through this investigation will not only help improve the current understanding of consumers’ health information search behavior, but will also shed light on how search systems can be tailored to individual consumers’ information preferences and needs.

## Methods

### Participants

Participant observation in an experimental setting was used for data collection. This method is appropriate because, in order to examine the effects of preference for information on participants’ behavior while they interact with search engines, it is necessary to control the search tasks and systems used. A convenient sample of 40 participants who were general consumers of health information (ie, who had searched previously on the Web for health information) was recruited by a message sent to a mailing list of students, faculty, staff, and alumni at a large research university. The age of the 40 participants ranged from 18-55 (mean 25.0, SD 8.5), with 10% (4/40) being between the ages of 18 and 20, 78% (31/40) between 20 and 30, and 13% (5/40) between 30 and 55. Each participant was compensated with US$15.

### Platforms

**Figure 1:**
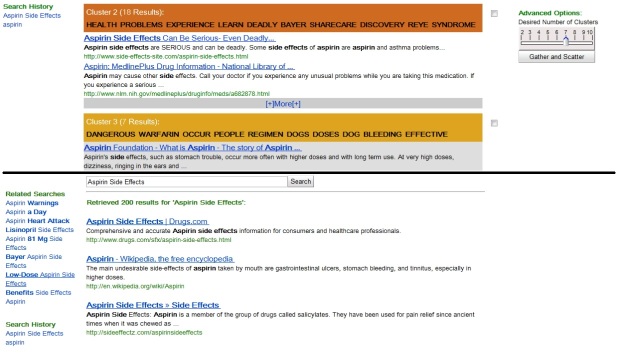
Screenshot of search results - the upper half of the image shows the scatter/gather interface and the lower half shows the classic interface.

The participants used one of two interfaces (Figure 1): (1) a classic Web search engine interface or (2) a Scatter/Gather-enabled search interface. Both interfaces are based on the Bing API (application programming interface); when a user types keywords into either interface, they are sent to Bing and the results are retrieved from Bing (Microsoft’s Web search engine). The classic interface resembles general Web search engines. It features a simple search box for keywords and the search results are presented as a relevance-ranked list. 

The Scatter/Gather interface has the same search box, but the results are grouped into clusters based on their topic similarities and the clusters are ranked by size (ie, the number of results contained in each cluster). A set of keywords is also presented along with each cluster [34]. The Scatter/Gather-enabled interface was chosen because its clustering function, as well as the keywords, may help to reduce the difficulty that consumers have with medical terminology.

### Tasks

Two types of tasks were defined: simple look-up and exploratory. Look-up tasks are tasks aimed at finding particular health-related facts, whereas exploratory tasks are oriented toward learning, investigating, and making sense of specific health issues. This classification was adopted because it can effectively characterize the goal and complexity of a health information request [35]. Four simulated search tasks were created—two look-up and two exploratory tasks—as shown in Table 1.

To ensure that the tasks reflected those that general health information consumers would search for in real life, the first three tasks were derived from questions posted in the health section of “Yahoo! Answers”, a social Q&A site where consumers post questions and/or answer questions posted by peers [36]. The last task was adapted from a task set designed for testing a Medline-based medical document collection [37]. In addition, in two pilot sessions, we asked participants to comment on the tasks. Both agreed that the tasks were something that they were likely to do in real life.

**Table 1:** Search tasks.

Task	
**Look-up tasks**	
	A friend of yours is an athlete. Now he wants to increase his muscle mass. He has been training without creatine, but would like to start a regimen. He is seeking your advice on this. You decide to find out what the side effects of taking creatine are.
	A heart attack is a medical emergency and prompt treatment increases the chance for survival. According to the American Heart Association, heart attacks cause 1 out of every 5 deaths. According to the National Institutes of Health (NIH) more than 1.2 million heart attacks occur each year in the United States and about 460,000 of these are fatal. Approximately 300,000 people die annually from heart attacks before they can receive medical treatment. To be prepared for possible emergencies, you decide to find out what to do when a person around you has a heart attack.
**Exploratory tasks**	
	Imagine that one of your close family members has lived with diabetes for years. Recently, he was also diagnosed with hypertension. You decided to do some research on the clinical associations between the two conditions so that you are able to effectively discuss with him about various implications of this diagnosis.
	Imagine that you recently began suffering from migraines. You heard about two possible treatments for migraine headaches, beta-blockers, and/or calcium channel blockers, and you decided to do some research about them. At the same time, you want to explore whether there are other options for treating migraines without taking medicines.

### Data Collection Procedure

The data collection took place in a private lab. Upon arrival, a moderator gave the participants an overview of the study and asked each participant to read and sign an informed consent form. After this, the participants completed a questionnaire reporting demographics, as well as their experience with Web and health information searches. Then they were asked to complete the MBSS and the KHOS-I scales. The MBSS consists of four hypothetical stress situations, each of which is followed by eight declarative statements, with four reflecting monitoring behavior and four blunting behavior. Participants were instructed to check all the statements that applied to them [11]. The KHOS-I consists of seven items and measures people’s desire to be informed and their desire to gather information. Participants rated their answers in a forced-choice format as “agree” or “not agree.” Higher scores indicated a greater need for seeking information on issues regarding their health [12]. The two measures have been used in a number of studies and demonstrated acceptable reliability and validity [6,8].

After completing the two scales, participants were randomly assigned to one of the two interfaces. As a result, 20 participants used the classic search interface and 20 used the Scatter/Gather interface. Each participant, using one or the other interface, completed all four tasks. The order in which the tasks were presented was randomized to reduce learning effects. Before the search began, participants watched a video tutorial demonstrating the basic functions of the interface to which they were assigned. During the search, when they checked out a website from the results list, they were prompted to rank the relevance and usefulness of the site on a 7-point Likert scale (1=not relevant, 7=relevant; 1=not useful, 7=useful). The ratings, along with the search queries and websites visited, were logged automatically by the search systems.

After completing each task, participants completed a short questionnaire assessing the difficulty of the task, the mental effort it required, and their level of satisfaction with their performance on a 5-point Likert scale. Camtasia software recorded each search session in video format. After completing all four tasks, participants filled out a questionnaire assessing their overall experience with the system that they had used. The questionnaire consisted of statements about users’ perceptions of the ease of use and usefulness of the system, their understanding of how the system worked, their levels of enjoyment and engagement, and their intentions toward using the system in the future. The rating scale was a 5-point Likert scale (1=strongly disagree with the statement, 5=strongly agree). The items measuring the ease of use and usefulness were adapted from prior studies [38,39]. The remaining aspects were each measured by a single item. At the end of the session, participants were asked to comment on search tasks and their behavior of performing the tasks. Each session lasted 1-1.5 hours.

### Data Analysis

The independent variable was preference for information, measured by the MBSS and the KHOS-I. For the MBSS, separate monitor and blunter scores were calculated [11]. Each participant’s monitoring score was determined by adding the number of monitoring items checked (M) and the blunting score was determined by adding the number of blunting items checked (B). The Kuder-Richardson Formula 20 test [40] was used to examine the internal consistency of the two subscales. The results suggest that neither subscale was reliable with participants in this study (Monitoring subscale: Cronbach alpha=.53; Blunting subscale: Cronbach alpha=.35). As a result, no further analyses were performed based on the MBSS measurements, similar to what has been done in previous research [27,41].

The KHOS-I score was determined by adding the number of items participants checked indicating a desire for information. The Kuder-Richardson 20 coefficient for KHOS-I was 0.77, indicating an adequate internal consistency for this scale with participants in this study. Thus, further analyses were performed based on the KHOS-I scale. Descriptive statistics showed that the mean score of the KHOS-I for the sample in this study was 4.3 (SD 2.1). Participants were categorized into two groups using a median split [42]: those scoring above the median were in the high-preference group and those scoring equal to or below the median were in the low-preference group. The two groups differed significantly on the KHOS-I score (*t*_38_=7.7, *P*=.001).

The dependent variables included the participants’ search behavior, their perceptions of the tasks and task performance, and their experience with search systems. Search behavior was operationalized by typical actions involved in a search process [32,43], including (1) session length, (2) query behavior (the number of queries submitted, query length, and query reformulation), and (3) accessing of results (the number of sites viewed, the number of sites rated as relevant, and the number of sites rated as useful; a site is considered relevant or useful when the rating is greater than 4 on a 1-7 Likert scale). These data were recorded in transaction logs. The analysis of query reformulation followed the topology developed by Rieh and Xie [44] and modified by Zhang et al [45]. The resulting topology included five types of query reformulations based on semantic changes: (1) specification (participants specify the meaning of the previous query), (2) generalization (participants generalize the meaning of the previous query), (3) parallel movement (participants replace one concept in the previous query with a new concept and the two queries have a partial overlap in meaning), (4) new concept movement (participants change to new concepts and the new query does not overlap with the previous query), and (5) rephrasing (participants rephrase the previous query by changing the form of the query without changing the meaning, such as correcting misspellings and rephrasing the previous query into a question). Two independent coders, the author and a trained graduate student, coded all the query reformulation instances; the inter-coder reliability was 98.5%. Discrepancies were resolved by discussion.

The participants’ perceptions of the search tasks and task performance were measured by their ratings on task difficulty, mental effort, and task performance after completion of each task. Their experience with the search system was measured by ratings on the user experience questionnaire administered at the end of each search session. These data were imported into SPSS for statistical analysis.

A series of *t* tests suggests that the two interface groups did not differ in demographics (including age, gender, computer experience, and health information search experience), nor in any of the measurements on search behavior, perceptions of tasks, and experience with search systems [45]. In addition, two-way ANOVAs (analyses of variance) suggested that there were no significant interactions between interfaces and information preferences (as measured by KHOS-I). Because the focus of this paper is on the impact of preference for information, to simplify the presentation, the two interface groups were pooled together for further data analysis. Then, *t* tests were used to investigate the impact of preference for information. The level of statistical significance was set at .05.

## Results

### Characteristics of the Participants

Table 2 summarizes the demographics of the two information preference groups—high-preference and low-preference—as well as their KHOS-I scores and their experience with health information searches. A chi-square test indicated that the two groups did not differ by gender (χ^2^_1_=1.44, *P*=.31). As well, *t* tests indicated that they also did not differ in age, Web experience, or experience with health information searches (years and frequency of searching for health information). In addition, all of the participants had or were in the process of getting a college degree and the two groups did not differ in their education levels.

**Table 2:** Demographics and health information search (HIS) experience by preference for information.

	Low preference	High preference	*t* (df)	*P* value
KHOS-I score, mean (SD)	3.0 (1.7)	6.6 (0.5)	−7.72 (38)	<.001
Male	8	7	-	-
Female	18	7	-	-
Age (years), mean (SD)	24.9 (8.0)	25.4 (9.6)	−0.18 (38)	.86
Web experience (years), mean (SD)	12.5 (4.3)	14.6 (4.7)	−1.47 (38)	.15
HIS experience (years), mean (SD)	3.9 (1.2)	3.2 (1.6)	1.57 (38)	.12
HIS frequency (times/month), mean (SD)	2.9 (0.7)	2.6 (1.1)	0.71 (38)	.48

### Information Search Behavior

#### Overview

Participants’ behavior while interacting with search engines was measured in light of three aspects: session length (task completion time), query formulation, and accessing of results.

#### Task Completion Time

Table 3 summarizes the two groups’ mean task completion times for each type of task. The *t* tests indicated that preference for information did not have an impact on the task completion time for either the look-up or exploratory tasks.

**Table 3:** Task completion times (in seconds).

	Low preference Mean (SD)	High preference Mean (SD)	*t* (df)	*P* value
Look-up	452.4 (186.0)	382.4 (125.9)	1.26 (38)	.22
Exploratory	556.6 (211.9)	549.3 (151.7)	0.12 (38)	.91

#### Query Formulation

Table 4 shows the average number of queries submitted by the two groups in completing each type of task and the average query length. The *t* tests indicated that the two groups differed only in the average length of queries submitted to solve look-up tasks, with the low-preference group submitting significantly longer queries than the high-preference group (*t*_38_=2.42, *P*=.02).

**Table 4:** Number of queries submitted and query length.

		Low preference Mean (SD)	High preference Mean (SD)	*t* (df)	*P* value
**Look-up**					
	No. of queries	2.1 (1.4)	2.4 (1.0)	−0.67 (38)	.51
	Query length^a^	5.2 (1.6)	4.1 (1.1)	2.42 (38)	.02
**Exploratory**					
	No. of queries	3.4 (1.5)	3.8 (1.8)	−0.80 (38)	.43
	Query length^a^	3.5 (1.0)	3.8 (0.8)	−0.85 (38)	.40

^a^Number of search words/terms

**Table 5:** The patterns of query reformulation.

		Low preference, % Mean (SD)	High preference, % Mean (SD)	*t* (df)	*P* value
**Look-up**					
	Specification	18.4 (30.0)	26.7 (28.8)	−0.84 (38)	.40
	Generalization	7.7 (21.5)	5.9 (15.4)	0.27 (38)	.79
	Parallel movement	19.4 (29.0)	33.2 (29.4)	−1.43 (38)	.16
	New concept movement	3.9 (9.4)	4.4 (11.3)	−0.17 (38)	.87
	Rephrasing	19.8 (36.8)	8.3 (21.4)	1.07 (38)	.29
**Exploratory**					
	Specification	26.8 (23.4)	24.8 (20.2)	0.27 (38)	.79
	Generalization	13.2 (15.9)	19.3 (27.6)	−0.89 (38)	.38
	Parallel movement	16.9 (18.9)	32.9 (27.6)	−2.18 (38)	.04
	New concept movement	35.1 (29.7)	11.6 (20.0)	2.65 (38)	.01
	Rephrasing	8.0 (14.3)	11.3 (18.0)	−0.64 (38)	.53

Table 5 shows the patterns of query reformulation. Because participants differed in the number of queries they submitted, percentages were used to normalize the data for comparisons of their behavioral patterns. The *t* tests indicated that the two groups did not differ in their query reformulation behavior when completing the look-up tasks. In contrast, when completing the exploratory tasks, the high-preference group performed a significantly higher percentage of parallel movements (*t*_38_=2.18, *P*=.04) and a significantly lower percentage of new concept movements (*t*_38_=2.65, *P*=.01).

#### Accessing of Results

Three aspects of the access of results were examined: the number of sites viewed by the participants, the percentage of the sites rated as relevant, and the percentage of the sites rated as useful. Table 6 shows the statistics. The *t* tests indicated that the two groups accessed an equal number of results in solving both types of tasks and they also reported equal percentages of sites as relevant and useful.

**Table 6:** Number of sites visited, the percentage of sites rated as relevant, and the percentage of sites rated as useful.

		Low preference Mean (SD)	High preference Mean (SD)	*t* (df)	*P* value
**Look-up**					
	No. of results	4.2 (2.4)	3.7 (1.8)	0.69 (38)	.50
	Relevant (%)	80.3 (20.6)	83.4 (21.5)	−0.44 (34)	.66
	Useful (%)	70.2 (27.8)	71.4 (21.2)	−0.13 (35)	.90
**Exploratory**					
	No. of results	4.8 (2.1)	5.1 (2.0)	−0.33 (38)	.75
	Relevant (%)	84.2 (18.1)	72.3 (25.9)	1.68 (36)	.10
	Useful (%)	73.1 (24.0)	57.9 (27.4)	1.79 (36)	.08

### Perceptions of Tasks and Task Performance

Table 7 shows the two groups’ perceptions of task difficulty, the mental effort required to complete the tasks, and their satisfaction with their performance. The *t* tests indicated that the two groups did not differ in their perceptions of simple look-up tasks, but one difference was found for exploratory tasks: the high-preference group perceived the exploratory tasks to be more difficult than the low-preference group (*t*_38_=2.01, *P*=.05).

**Table 7:** Perceptions of task difficulty and task performance.

		Low preference Mean (SD)	High preference Mean (SD)	*t* (df)	*P* value
**Look-up**					
	Task difficulty^a^	2.1 (0.6)	2.0 (0.6)	0.69 (38)	.50
	Mental effort^b^	2.4 (0.7)	2.4 (0.6)	−0.11 (38)	.91
	Satisfaction^c^	4.2 (0.4)	4.1 (0.6)	0.52 (38)	.60
**Exploratory**					
	Task difficulty^a^	2.3 (0.4)	2.6 (0.6)	−2.01 (38)	.05
	Mental effort^b^	2.6 (0.5)	2.6 (0.6)	0.04 (38)	.97
	Satisfaction^c^	4.0 (0.5)	4.0 (0.7)	0.20 (38)	.85

^a^Rated on a 5-point scale (1-very easy, 5-very difficult)

^b^Rated on a 5-point scale (1-very small amount, 5–very large amount)

^c^Rated on a 5-point scale (1-very disappointed, 5–very satisfied)

### User Experience With the Systems

Table 8 shows the two group’s experience with the search systems used in the study. The *t* tests indicated that the two groups differed in their perceptions of the systems’ usefulness, with the low-preference group rating the search systems as more useful (*t*_38_=2.18, *P*=.04).

**Table 8:** User experience^a^.

	Low preference Mean (SD)	High preference Mean (SD)	*t* (df)	*P* value
Ease of use	4.3 (0.5)	4.2 (0.6)	0.47 (38)	.64
Usefulness	4.1 (0.6)	3.5 (0.9)	2.18 (38)	.04
Understand how it works	3.8 (0.7)	3.6 (0.8)	0.59 (38)	.56
Enjoyment	3.8 (0.9)	3.6 (0.9)	0.70 (38)	.49
Engagement	3.3 (0.8)	3.2 (1.2)	0.29 (38)	.77
Future use	3.5 (0.9)	3.6 (1.1)	−0.50 (38)	.62

^a^Rated on a 5-point scale (1-strongly disagree, 5-strongly agree)

## Discussion

### Summary

This study explores the impact of a personality factor, preference for information, on the health information search behavior of general consumers, on their perceptions of tasks and performance, and on their experience with search systems. In prior studies, preference for information was mainly investigated in the context of patient-provider interactions, in order to predict patients’ behavior of seeking information from providers and to inform interventions that can help patients cope with stress [30,46]. This study makes a contribution by going beyond this traditional context to the context of consumers’ interaction with Web-based search engines for health information. This extension is important since searching for health information has become one of the most popular online activities [47] and information found online has an increasingly significant impact on consumers’ health care decisions [1]. The discussion is organized around two themes: (1) the measurement of preference for information, and (2) the impact of preference for information on consumers’ health information search behavior and the implications for system design. The limitations of this study are also discussed.

### Measurement of Preference for Information

Participants’ preference for information was measured by the MBSS and the KHOS-I. Both the monitoring and blunting subscales had poor internal consistency, indicating low reliability of the MBSS in measuring consumers’ tendency to seek or to avoid information in the context of searching for health information in the Web environment. Several factors may contribute to this result. First, the sample size was small. The study involved only 40 participants. Second, the MBSS has inherent limitations. As critiqued by other researchers, scenarios in the MBSS are hypothetical. Particularly, the scenario of “being held hostage by a group of armed terrorists” is too far removed from most people’s life experiences [48,49]. Moreover, the validity of the scale has been questioned [50]. For example, Barsevick and Johnson [20] found that the MBSS was not a sensitive indicator of preference for information for patients undergoing colposcopy. A third factor may be the nature of the context of this study. In most prior studies, the MBSS was applied to patients in life-threatening medical situations (eg, cancer and heart disease) and/or undergoing stressful medical procedures (eg, colposcopy and biopsy) [13,20,27]. In this study, participants were general health information consumers and the scenarios were of an everyday nature and less life-threatening. The applicability of the MBSS in predicting people’s preference for information in such a context merits more investigation as more and more consumers go online for health information.

In contrast, the KHOS-I had a sufficient level of reliability (Cronbach alpha=.77). This might have been because the KHOS-I was initially designed to measure individuals’ tendency to seek information in routine and general health care contexts, which is well reflected in the statements in the scale, for example: “I usually ask the doctor or nurse lots of questions about the procedures during a medical exam.” Similar levels of reliability of this scale were found not only for groups with specific conditions, such as cancer, [9,51], myocardial infarction [52], and dental problems [46], but also for general health information consumers, such as undergraduate students [12]. In this study, the mean KHOS-I was 4.3 (scale 0-7), indicating that participants in this study had a comparatively high preference for information, which may be accounted for by the participants’ young age and their overall high level of education.

We also found that the MBSS and the KHOS-I were not correlated, which is consistent with two earlier studies [20,49]. One potential explanation for this is the low reliability of the MBSS for the participants in this study. The other reason could be that the two scales may well measure different constructs [20].

### The Impact of Preference for Information on Consumers’ Search Behavior, Perceptions of Tasks, and Experience With Search Systems

Information searching involves three major elements: the user, the task, and the system [32]. To understand the impact of preference for information on health information searches, we measured participants’ search behavior (session length, query formulation, and accessing of results) and assessed their perceptions of tasks (for difficulty, mental effort, and performance) and their experience with the search systems used in the study (for ease of use, usefulness, understanding of the systems’ working mechanisms, enjoyment, engagement, and projected future use of the system).

Participants with different levels of preference for information differed significantly on several of these measurements. First, the length of queries differed. The average length of queries submitted by the high-preference group was significantly shorter than those submitted by the low-preference group when completing the look-up tasks. A possible explanation is that participants with a low preference for information were eager to get the right answer as quickly as possible, so that they attempted to make the search queries as specific as possible, whereas the participants with a high preference were willing to do some exploration and thus submitted more general queries. An examination of the actual queries revealed that many queries submitted by the low-preference group were complete questions (eg, “what to do if someone has a heart attack”), rather than keywords, which further supports this speculation.

A second difference was the pattern of query reformulation. When completing the exploratory tasks, the high-preference group made a significantly higher percentage of parallel movements than did the low-preference group, whereas the low-preference group made a significantly higher percentage of new concept movements than the high-preference group. In parallel movements, participants replace a concept in the previous query, so that the two queries have partial overlap in meaning, and in new concept movements, participants change to a new concept and the new query does not overlap with the previous query. This result suggests that, when exploring a health-related topic, people with a high preference for information may be more likely to take steps to gradually explore relationships between concepts, whereas those with a low preference may be more likely to investigate concepts one by one. This result further indicates that people with a high preference for information might develop a more complex mental representation of the medical problem at hand, whereas people with a low preference might simplify the problem and thus develop a comparatively less complex conceptual representation. Prior studies on patient-provider interactions have revealed that patients with a high preference for information often ask more questions of the provider [12,15,20]. However, few studies investigated the nature of the questions, how the questions were related to one another, and whether more concepts were involved in questions imposed by high-preference patients. In future studies, qualitative studies are needed to shed light on these research questions, which will also help interpret the results of this study.

Along the same lines, the finding that patients with a high preference for information tended to ask more questions of providers naturally leads to an expectation that when searching for health information, particularly with exploratory tasks, high-preference participants may submit more queries to the system and visit more search results than their low-preference counterparts. However, such results were not observed in this study. A possible explanation is the experimental nature of the study. Participants were performing assigned tasks, rather than their own tasks. A naturalistic approach to data collection, such as transaction log analysis, would help elucidate the relationships between preference for information and the number of search queries and search results visited. Another explanation is that high-preference participants, more so than low-preference patients, may possess a greater ability to process retrieved information. Therefore, they did not submit more queries and examine more results, but acquired more information. Future studies may test this speculation by comparing the learning outcomes of low- and high-preference groups after a search session.

A third difference between the groups was the participants’ perceptions of task difficulty. The high-preference group perceived the exploratory tasks to be significantly more difficult than did the low-preference group. This relationship did not hold with look-up tasks, which involved seeking factual information. This finding may also be attributed to the possibly more complex mental representations that the high-preference group developed for the exploratory tasks.

The fourth difference was the participants’ perceptions of the usefulness of the search systems. The high-preference group perceived the systems to be less useful than did the low-preference group, which seems consistent with their perceptions of the difficulty of exploratory tasks. It is possible that the high-preference group’s perceptions of the task difficulties made them less satisfied with the utility of the systems for addressing their needs. Comparable results have been found in the context of patient-provider interactions. For example, Timmermans et al [15] reported that, in cancer treatment with a palliative intention, high monitors reported having more doubts about the treatment decision and being less satisfied with the information received, while high blunters expressed fewer doubts and more satisfaction.

These results indicate that preference for information has an impact on consumers’ interactions with search systems for health information. As reviewed, prior studies have consistently suggested that information interventions in patient-provider encounters are most effective when they are congruent with receivers’ preference for information [53]. It is natural to postulate that health information search systems may be most useful and effective when they are tailored to individuals’ information preferences. Some system design implications can be drawn from the results of this study. For example, for those with a low preference for information, the system could provide a “natural” user interface that allows them to write queries in natural language (ie, long queries or queries in question format) rather than artificial keywords [54], to accommodate their need for imposing very specific queries. At the same time, the system should improve its ability in processing long queries [55]. When presenting results, the system could present the most specific query results at the top of the results list or recommend a list of more specific queries. For those with a high preference, the system could allow them to explore relationships between concepts and recommend new but related concepts (based on medical thesauri or on the mining of query logs) to accommodate their propensity to develop complex networks of concepts. When recommending queries, both more general and more specific queries can be provided to allow flexibility in exploration. Moreover, systems can offer functions to present search results in visual ways that can enable users to explore relationships among the concepts involved in the results (eg, a tree-map view or a network view of concepts). In this study, the Scatter/Gather interface clustered results based on topic similarity and provided a set of keywords to represent each cluster, but failed to illustrate relationships between concepts. This may be one of the reasons that Scatter/Gather interface did not differ from the basic search interface in supporting searches.

### Limitations and Future Studies

There are limitations to this study. First, the sample consisted primarily of people with high education and high computer literacy; thus, the generalizability of the results is limited. Future studies should extend the sample to people with low computer and health literacy, as well as to patients with particular conditions, such as cancer and diabetes. Such studies can inform the tailoring of information systems to the needs of underserved groups. Second, a limited number of search behavior variables was measured, which directly limits our understanding of the scope of the impact of preference for information on consumers’ health information search behavior. In future studies, researchers should examine consumer behaviors in relation to, for example, the content examined (eg, evidence-based medical research vs user-generated content) and the types of sites visited (eg, commercial sites vs academic sites), to examine whether preference for information has an impact. Third, the tasks used in this study were classified as look-up and exploratory tasks. Future studies can look into other ways of classifying health-related search tasks, such as by the goals of searches (eg, seeking diagnosis, treatment, or medical facilities) [56]. In addition, only four search tasks, two in each category, were involved in this study. Future studies could attempt to include a larger number of search tasks to further reduce the possible impact of other task features, such as the topic, on people’s search behavior.

### Conclusions

The personality trait, preference for information, showed an impact on general consumers’ search behavior for health information, their perceptions of task difficulties, and their experience with search systems. Compared to people with a low preference for information, those with a high preference exerted greater efforts in information searching. These efforts were manifested not so much at the behavioral level (eg, submitting more search queries or checking out more search results), but more at the conceptual level, with those with a high preference being more likely to use more general queries when searching for specific factual information and to develop more complex mental representations of health concerns of an exploratory nature and try different combinations of concepts to explore these concerns. Consequently, high-preference users were also more demanding on the system. These findings suggest that system developers should take into consideration users’ preference for information in designing health information search systems. To further advance our knowledge about consumers’ health information search behavior and to inform the design of more effective systems, the influence of other personality factors, such as locus of control, on information searches should also be examined.

## Abbreviations

KHOS-I: Krantz Health Opinion Survey-Information scale
MBSS: Monitor-Blunter Style Scale
